# Toxicological Assessment of Particulate and Metal Hazards Associated with Vaping Frequency and Device Age

**DOI:** 10.3390/toxics11020155

**Published:** 2023-02-07

**Authors:** Jennifer Jeon, Qian Zhang, Patrick S. Chepaitis, Roby Greenwald, Marilyn Black, Christa Wright

**Affiliations:** 1 Chemical Insights Research Institute, UL Research Institutes, Marietta, GA 30067, USA; 2 School of Public Health, Georgia State University, Atlanta, GA 303132, USA

**Keywords:** metals, particles, e-cigarettes, genotoxicity, vaping, aerosols, exposures

## Abstract

Electronic nicotine delivery systems (ENDS) aerosols are complex mixtures of chemicals, metals, and particles that may present inhalation hazards and adverse respiratory health risks. Despite being considered a safer alternative to tobacco cigarettes, metal exposure levels and respiratory effects associated with device aging and vaping frequency have not been fully characterized. In this study, we utilize an automated multi-channel ENDS aerosol generation system (EAGS) to generate aerosols from JUUL pod-type ENDS using tobacco-flavored e-liquid. Aerosol puff fractions (1–50) and (101–150) are monitored and sampled using various collection media. Extracted aerosols are prepared for metal and toxicological analysis using human primary small airway epithelial cells (SAEC). ENDS aerosol-mediated cellular responses, including reactive oxygen species (ROS), oxidative stress, cell viability, and DNA damage, are evaluated after 24 h and 7-day exposures. Our results show higher particle concentrations in later puff fractions (0.135 mg/m^3^) than in initial puff fractions (0.00212 mg/m^3^). Later puff fraction aerosols contain higher toxic metal concentrations, including chromium, copper, and lead, which elicit increased levels of ROS followed by significant declines in total glutathione and cell viability. Notably, a 30% increase in DNA damage was observed after 7 days because of later puff fraction exposures. This work is consistent with ENDS aerosols becoming more hazardous across the use of pre-filled pod devices, which may threaten respiratory health.

## 1. Introduction

Since their emergence nearly 30 years ago, electronic nicotine delivery systems (ENDS) format features have evolved to enhance the user’s vaping experience. While ENDS designs have matured, their common basic components, including a cartridge, atomizer, battery, and mouthpiece, have been maintained [1]. The cartridge is a reservoir for e-liquid, which consists of vegetable glycerin (VG), propylene glycol (PG), nicotine, and flavoring agents [2]. Another important component is an atomizer, which contains a heating coil that connects to a battery providing the necessary power to vaporize the e-liquid into an aerosol that is inhaled via the mouthpiece [1]. Based upon these basic components, each ENDS generation has its own additional unique features including different battery capacities, design of the devices, modifiability, and types of nicotine used [1]. Of the many commercially available ENDS formats, the pod-type is considered a fourth-generation ENDS with different battery styles that allows automated fixed voltage delivery and coil temperature maintenance [1,3,4]. Pod-type ENDS have gained popularity among adolescents because of their simple design and the easy access to disposable pre-filled pods [3,5]. In fact, the 2020 National Youth Tobacco Survey data showed a prominent preference for pod-type ENDS among adolescents, with 49.5% usage prevalence [4,6]. Despite the increasing popularity of the pod-type ENDS, the impact on respiratory health remains unknown because of numerous variables that can alter aerosol properties and levels of exposure. 

As the popularity of the pod-type ENDS device gradually increases, safety concerns have escalated, warranting further examination of the hazards posed by pod-type ENDS-emitted aerosol exposures. Indeed, differential vaping patterns or levels of ENDS usage can contribute to exposure variances to various organic and inorganic hazards, including heavy metals [7,8,9]. Several metals have been detected in pod-type ENDS aerosols, such as chromium (Cr), nickel (Ni), copper (Cu), zinc (Zn), cadmium (Cd), tin (Sn), manganese (Mn), and lead (Pb) [10,11]. The primary sources of heavy metals are the metallic components, such as filaments and coils, which can degrade when in contact with oxidized acidic e-liquids leading to additional metal exposure [10]. For instance, a recent study has reported a higher level of Ni and Cr concentration in the aerosolized format compared to the non-aerosolized e-liquid because of leaching from the heating element [12]. Furthermore, a significant increase in Ni level was reported after heavy use of ENDS devices, highlighting the necessity to evaluate potential respiratory health effects across device lifetime [13]. Increased metal exposure is a critical risk factor for various respiratory diseases, such as respiratory inflammation, asthma, COPD, and respiratory cancer [14,15,16]. However, there is a lack of evidence to determine the level of metal exposure from the pod-type ENDS aerosol because of the inconsistency of aerosol generation parameters, type of device, and e-liquid selection in recent studies [7,17,18]. Therefore, a detailed evaluation of the level of metal exposure derived from pod-type ENDS usage is needed to determine the potential adverse effects on respiratory health. The hazardous components within ENDS aerosols, such as heavy metals and particulate matter, can impair airway epithelial cells, which provide critical barrier protection in the respiratory system [19,20]. According to recent studies, the varying sizes of ENDS-emitted particles that are smaller than 2.5 µm can penetrate lower airways and increase the risk for small airway diseases [21,22]. Once hazardous particulates penetrate and deposit within the respiratory system, airway epithelial cells can activate inflammatory responses by releasing pro-inflammatory cytokines, such as IL-6 and IL-8, leading to epithelial barrier dysfunction [23,24]. Several studies have highlighted the toxicological effect of ENDS aerosols on respiratory health, including heightened inflammatory responses, oxidative stress, and DNA damage [25,26,27]. For example, a dose-dependent correlation between the level of ENDS aerosol exposure and cellular inflammation has been reported in several in vitro studies, along with the elevated reactive oxygen species (ROS) generation, which are both mechanisms leading to cell cytotoxicity [28]. Specifically, heavy metals that are highly present in ENDS aerosol, such as Cr, Cu, and Al, are reported to disrupt several cellular processes, such as aerobic respiration, resulting in ROS production and oxidative stress [29,30,31]. Extensive levels of oxidative stress can cause DNA oxidation, strand breakage, and base modification, which further leads to genomic instability in airway epithelial cells [1,32]. Indeed, vaping can lead to extensive DNA structural modifications in airway epithelial cells, causing irreversible parenchymal lung tissue damage, altered gas exchange, and lung function impairment [33]. However, there is still a gap in knowledge because of the differences in study designs, exposure scenarios, type of ENDS device, and puffing parameters within current studies. Therefore, a comprehensive evaluation should be conducted to analyze and identify the parameters that influence adverse health effects that are due to ENDS product usage. 

This study aims to address the following experimental questions. (1) What are the aerosol concentration and metal composition of each puff fraction? (2) What are the toxicological effects of each puff fraction on respiratory health? Within this study, aerosol characterization was performed to measure particle size distribution and physicochemical properties using scanning mobility particle sizer (SMPS) and inductively coupled plasma mass spectrometry (ICP-MS), respectively. Cellular bioassays such as CellROX^®^, total glutathione (GSH), cell proliferation assay (MTS), and CometChip^®^ were performed to measure reactive oxygen species (ROS) generation, oxidative stress, cellular viability, and DNA damage, respectively. The findings reported herein may be used to provide guidance to ENDS users on safer vaping patterns or behaviors resulting in lower respiratory health risks.

## 2. Materials and Methods

A custom-built four-channel ENDS aerosol generation system (EAGS) was purchased from IES Technologies (Morgantown, WV, USA), and the fluorinated ethylene propylene (FEP) condensation tube was purchased from Savillex (Eden Prairie, MN, USA). The JUUL^®^ device and JUUL^®^ Virginia Tobacco 5% e-liquid pod were utilized and evaluated in this study. To evaluate the adverse cellular effects of ENDS aerosol exposure, primary small airway epithelial cells (SAEC, PCS-301-010™, ATCC, Manassas, VA, USA) were purchased from ATCC (Manassas, VA, USA) and cultured by using PneumaCult™Ex Plus Medium (STEMCELL, Vancouver, BC, Canada) and PneumaCult™-ALI media (STEMCELL Vancouver, BC, Canada). For the toxicological assays, the CellROX^®^ Orange ROS Detection reagent (Invitrogen, Waltham, MA, USA), GSH-Glo^TM^ Glutathione reagent (Promega, Madison, WI, USA), and CellTiter 96 Aqueous One Solution (Promega, Madison, WI, USA) were used for the reactive oxygen species (ROS) detection, total glutathione level measurement, and cellular viability measurement, respectively. SybrGold nucleic acid stain (Invitrogen) was purchased from Invitrogen to stain SAEC nuclei and image the cells using the Cytation 1 multi-mode imaging system (Biotek, Winooski, VT, USA). The 2% nitric acid solution and other reagents used for ICP-MS analysis were purchased from ARISTAR^®^ PLUS and VWR Chemicals BDH^®^.

### 2.1. ENDS Aerosol Generation, Characterization and Sample Collection

A custom-built four-channel automatic ENDS aerosol generation system (EAGS, IEStechno, Morgantown, WV, USA) was used to generate aerosols in a 6 m^3^ exposure chamber. A pod-type ENDS device (JUUL^®^) was selected for the aerosol sample collection device with Virginia-tobacco-flavored e-liquid at 5% nicotine strength. The pod-type ENDS (JUUL^®^) connected to EAGS was automatically operated using a pneumatic actuator, which was set to produce 55 mL of each puff with 3 s duration and an inter-puff interval of at least 30 s following the Center for Scientific Research Relative to Tobacco (CORESTA) Recommended Method No. 81 [34]. The generated ENDS aerosol was directed into the buffer chamber, located underneath the EAGS system, and diluted with clean air at a flow rate of 2.0 L/min. The thoroughly mixed aerosol from the buffer chamber was directed through a seven-port manifold and collected in two of the connected fluorinated ethylene propylene (FEP) condensation tube traps, and the rest was emitted into the chamber for particle monitoring [35]. The aerosol samples were collected at two different stages to compare the device aging effect during the sample generating process. The initial sample was collected during puffs 1–50, and the second sample during puffs 101–150. A scanning mobility particle sizer (SMPS, models TSI 3080 and 3786) was used to determine the aerosol size distribution and concentration for sizes ranging from 7–300 nm and an optical particle sizer (OPS, model TSI 3330) was used for sizes ranging from 0.3–10 µm. Details of EAGS and exposure chamber setups and aerosol characterization methods were previously described [36]. Importantly, the disposable pod-type ENDS (JUUL^®^) utilized in this study contain sufficient e-liquid for 200 puffs. However, our optimization studies revealed that only 150 puffs were attainable because of the pod format, and therefore, this was used as the upper puff limit.

### 2.2. FEP Tube Condensation Trap Preparation and Sample Extraction 

The pod-type ENDS (JUUL^®^)-emitted aerosol was collected in the 1800 cm fluorinated ethylene propylene (FEP) condensation tube with an inner diameter of 3.97 mm (Savillex, Eden Prairie, MN, USA). Prior to every experiment, all the FEP tubes were prepared as 15-inch lengths and cleansed using a 1% hydrochloric acid and 2% nitric acid cleaning solution. A total of 24 mL of cleaning solution was inserted into the FEP tube using a 50 mL syringe, followed by sonication for 5 min at 25 °C to remove any chemical residues introduced during the manufacturing process. After cleaning, the FEP tube was rinsed twice with deionized and distilled (DI) water for 10 min, refilled with DI water, and sonicated for 5 min at 25 °C, then dried completely using a filtered air system for an hour, then weighed. Each labeled FEP tube was weighed when clean and completely dried to minimize the potential error for the mass calculation. The pre- and post-experiment weights of the e-liquid-filled pod (JUUL^®^) were also measured to determine the total sample mass collection percentage. After the FEP tubes and used pods were reweighed following the sample collection process, the total sample collection mass and percentage were calculated by comparing the post-experiment weight to the pre-experiment weights.

All FEP tubes were stored at 4 °C after the sample collection and transferred to the Georgia State University campus to be weighed and extracted. The condensed aerosol inside the FEP tubing trap was extracted using a 75% methanol extraction solution. Each FEP tube was capped and filled with 50 mL of extraction solution before the sonication process. The coiled FEP tubes were sonicated for 5 min at 25 °C. After the sonication process, the exudated sample was vacufuged at 60 °C with 1000 rpm for 12 h until the volume was reduced to 100 µL in preparation for toxicological assessment and metal analysis. 

### 2.3. In Vitro Dosimetric Considerations

The multiple-path particle dosimetry (MPPD) model was employed to determine the amount of inhaled particle matter deposited within the small airways. The stochastic lung model was chosen, followed by an oral-pharyngeal exposure pathway to mimic human ENDS usage. A more detailed overview of the MPPD parameters can be found in the supplementary document (Table S1). Aerosol concentration and geometric mean were inputted, and outputs of average deposited mass (µg) divided by the average deposited surface area (cm^2^) were used to determine the average deposited dose (µg/cm^2^). To convert the total deposited dose to an in vitro concentration, we multiplied the total deposited dose (µg/cm^2^) by the surface area of one well within a 24-well trans-well plate (0.33 cm^2^) and then divided by the total exposure volume (0.1 mL).

### 2.4. Elemental Characterization of E-liquids and ENDS Aerosols—ICP-MS 

A Perkin Elmer NexION 5000 ICP-MS fitted with a 1.5 mL sample loop was used for analysis. The full details of instrumental setup, method parameters, and calibration information may be found in the Supplemental Information Section (Tables S2 and S3). A performance check specified by the manufacturer recommendation was performed using a tuning solution to monitor the optimal instrumental conditions. For e-liquid sample analysis, the mass of the e-liquid (tobacco flavor, 5% nicotine, commercially available) analyzed by ICP-MS was approximately 300 mg and was prepared with a dilution of 2% nitric acid solution (ARISTAR^®^ PLUS for trace metal analysis, VWR Chemicals BDH^®^, Radnor, PA, USA) up to 50 mL as described in the previous metal analysis study by Mara and colleagues [37]. The aerosol samples were prepared by fully evaporating 10 mL of the 75% methanol solution using a centrifuge concentrator (Vacufuge Plus, Eppendorf, Hamburg, DEU) set at 60 °C for about 2 h. The dilute 2% nitric acid solution described previously was then added to the dried samples. Three replicates of each sample were analyzed in helium kinetic energy discrimination mode to minimize elemental species interferences within the quadrupoles. Briefly, the quality control measures used for this study included external and internal calibration and recoveries for internal standards were acceptable and ranged from 71% to 118%, with the average being 90% across all elements in the analytical sequence. All initial concentration calculations were performed within the instrument’s Syngistix software version 3.2. To convert e-liquid sample concentrations from the instrument-given response units of µg/L, values were multiplied by the final dilution volume. For details of quality control measures see the Supplementary Materials. Recoveries of internal standards ranged from 71–118%, with the average being 90% across all elements in the analytical sequence. All coefficients of determination for the elements of interest were greater than or equal to 0.98. The RSD values among three replicates of each element reported were less than or equal to 21% (except for Fe, Cd, and Tl). The limits of detection (LOD) were calculated within 30 days of the study.

### 2.5. SAEC Culture and Exposure 

Primary small airway epithelial cells (SAEC) (ATCC^®^ PCS-301-010™, Manassas, VA, USA) were cultured in the T-75 cell culture flask using PneumaCult™-Ex Plus Medium (STEMCELL, Vancouver, BC, Canada) and incubated at 37 °C until 70–80% confluency. For the pod-type ENDS (JUUL^®^) aerosol exposure, SAEC was subcultured into the trans-well inserts within a 24-trans-well cell culture plate at 10,000 cells/mL density. The subcultured cells were maintained by changing the media every 2–3 days for approximately 14–21 days until a monolayer was formed. Once the monolayer was formed, the cells were air-lifted by removing the media from the apical surface. During the air-lifted phase, the cells were maintained by changing the basolateral media (PneumaCult™-ALI media, STEMCELL, Vancouver, BC, Canada) every 2–3 days. Each exposure (blank, puffs 1–50, puffs 101–150) was administered directly onto the apical surface (100 µL) and incubated at 37 °C for 24 h and 7 days. During the 7-day exposure, all the exposures were changed every day, and the basolateral media (PneumaCult™-ALI media, STEMCELL, Vancouver, BC, Canada) was changed every 2–3 days. In this study, all cytotoxicity assays employed negative (untreated) and positive controls. For positive control, 0.1% hydrogen peroxide was administered onto the apical surface (100 µL) and incubated at 37 °C for 20 min prior to each assay. 

### 2.6. Reactive Oxygen Species (ROS) Detection 

ROS were detected using the CellROX^®^ Orange ROS Detection Assay (Invitrogen, Waltham, MA, USA), which uses cell-permeable CellROX^®^ dye that exhibits bright orange fluorescence when oxidized. After 24 h and 7 days of pod-type ENDS (JUUL^®^) aerosol exposure, SAEC cells were washed with phosphate-buffered saline (PBS). CellROX^®^ Orange reagent was added to each well and incubated at 37 °C for 60 min. After the stain was removed, 3% paraformaldehyde (fixative) (Invitrogen, Waltham, MA, USA) was added and incubated at 37 °C for 15 min. After the fixative reagent was removed, the nuclei counterstain, DAPI (Invitrogen, Waltham, MA, USA), was added for 30 min and removed, followed by two PBS washes. The fluorescence signal was detected and imaged using the Cytation 1 multi-mode imaging system (Biotek, Winooski, VT, USA). 

### 2.7. Total Glutathione Level Measurement

The total glutathione level in SAEC was detected using the GSH-Glo^TM^ Glutathione assay (Promega, Madison, WI, USA), a luminescent assay that detects and quantifies the level of glutathione in the cells. The pod-type ENDS (JUUL^®^) aerosols were added to SAEC and removed after 24 h and 7 days. After washing the monolayer of SAEC, 1:100 dilution of glutathione s-transferase and luciferin in GSH-Glo™ reagent was added and incubated at 37 °C for 60 min. After removing the reagent, luciferin was added and incubated at 37 °C for 30 min. Luminescence was detected and measured using a Cytation 1 multi-mode imaging system (Biotek, Winooski, VT, USA). 

### 2.8. Cellular Viability Evaluations

The metabolic capacity of exposed SAEC was measured using the MTS assay (CellTiter 96 Aqueous One Solution, Promega, Madison, WI, USA) to determine cellular viability after pod-type ENDS (JUUL^®^) aerosol exposure. The MTS assay is a colorimetric assay that examines the capacity of exposed cells to metabolize tetrazolium salt into formazan. After 24 h and 7 days of exposure, the 1:10 dilution of MTS reagent (Promega, Madison, WI, USA) in fresh media was added to SAEC and incubated at 37 °C for 1 h. After the incubation, the absorbance was measured using the Cytation 1 multi-mode imaging system (Biotek, Winooski, VT, USA) at 490 nm.

### 2.9. DNA Damage Assessments

The CometChip assay was used to detect pod-type ENDS (JUUL^®^) aerosol-mediated DNA damage in SAEC. The CometChip assay allows the detection of single-stranded DNA damage in individual cells or nuclei by measuring the percentage of electrophoresed DNA fragments in the “comet tail”. Pod-type ENDS (JUUL^®^) aerosol-exposed SAEC were trypsinized, then neutralized and transferred into CometChip microwells. Cells loaded into the CometChip were then lyzed using a strong alkaline buffer solution for 45 min, followed by 30 min electrophoresis process. The CometChips were then neutralized and stained with SybrGold nucleic acid stain (Invitrogen, Waltham, MA, USA) overnight. The SAEC nuclei were imaged and analyzed using the Cytation 1 multi-mode imaging system (Biotek, Winooski, VT, USA). The length and intensity of the comet tail were measured and calculated into percent tail DNA to quantify the level of DNA damage in SAEC after pod-type ENDS (JUUL^®^) exposure.

### 2.10. Statistical Analysis 

All the exposures were completed in triplicate throughout the study, and each assay trial was duplicated for the toxicological analysis. Data from each assay and exposure group, including puffs 1–50, puffs 101–150, blank sample, negative control, and positive control, were analyzed by one-way ANOVA followed by Bonferroni’s post hoc analysis, where the significance level of less than or equal to 0.05 was employed. A two-tailed *t*-test was performed to determine differences within particle concentration data, where a *p*-value less than or equal to 0.05 was deemed significant. All analyses were performed in GraphPad Prism software (Prism version 9.3.1).

## 3. Results

### 3.1. ENDS Aerosol Generation Parameter Analysis 

A significant difference in particle concentration levels was found between the two puff fractions evaluated (Table 1). Specifically, the particle mass emission increased from 0.00212 mg/m^3^ to 0.135 mg/m^3^ during the aging process. In addition, the particle size distribution also shifted toward larger-sized particles in the later puff fraction (101–150) compared to the initial puff fraction (1–50), revealing a significant increase (*p* < 0.05) in geometric mean diameter (GMD) from 0.715 µm (1–50) to 0.902 µm (101–150). However, the standard deviation of the particles emitted at the later puff fraction (101–150) was smaller (σ = 0.0272) compared to the initial puff fraction (1–50, σ = 0.322), showing a tighter size distribution.

### 3.2. ENDS Aerosol Sample Collection Efficiency Evaluation

For each puff fraction, an average of 1030 mg of e-liquid (JUUL^®^ Virginia Tobacco 5%) was vaporized. In puff fraction 1–50, a total of 126 mg of ENDS aerosol sample was collected from the filter (2.74 mg) and the FEP tube (124 mg). The total mass collected during the puff fraction of 101–150 was 221 mg, which was collected from both the filter (2.12 mg) and the FEP tube (219 mg). Compared to the mass collected from the initial puff fraction (1–50), 94.7 mg more sample mass was collected in the later puff fraction (101–150).

### 3.3. Inhalation Dosimetry of ENDS Aerosols

Table 2 illustrates the average total deposited mass, deposited surface area, and deposited dose of JUUL^®^ Virginia Tobacco 5% aerosol metrics employed in the MPPD2 software. These inputs were then utilized to determine the in vitro concentration, which resulted in 39.3 µg/mL for the earlier puff fraction and 49.6 µg/mL for later puff fractions.

### 3.4. Impact of Device Aging on ENDS Aerosol Metal Composition

Twenty-one heavy metals relevant to human health were monitored by ICP-MS analysis in JUUL^®^ Virginia Tobacco 5% e-liquid and the aerosol generated from pod-type ENDS devices (JUUL^®^) (Table 3). All elemental species except aluminum, rubidium, cadmium, silver, thallium, and uranium were detected in the e-liquid sample at varying levels. Arsenic, barium, and lead were detected at elevated levels from the background in both e-liquid and aerosol fractions. Zinc, nickel, chromium, and iron were identified at extremely high concentrations within the pod liquid, indicating the possibility that the coil could be a nichrome alloy, and it was inferred that most of the metals detected within the e-liquid were leached from the device. Vanadium, manganese, cobalt, and strontium, all considered heavy metals relevant to human health, were also present within the analyzed e-liquid and aerosols. The background metal levels of each Teflon filter examined within this study were below the limit of detection. 

Generally, the concentrations of metals were lower by volume in the puff fractions in comparison to the e-liquids, with the exception being aluminum. This result is more understandable in the context of metal volatility versus liquid extraction. Zinc amounts remained high within the aerosol puff fractions assessed, whereas iron levels were significantly lower between liquid and aerosol fractions. An unexpected finding was the presence of rubidium within the aerosol fractions because this element is not ubiquitous and has little commercial application. Additionally, iron was not detected in certain puff fractions above the LOD. There were significantly higher concentrations (*p* < 0.05) between the 1–50 and 101–150 puff fractions for aluminum, chromium, copper, rubidium, barium, and lead, and higher concentrations (not statistically significant) of vanadium, manganese, and arsenic, while cobalt, nickel, and zinc remained fairly stable among the aerosol portions. 

### 3.5. Reactive Oxygen-Species Generation in ENDS-Aerosol-Exposed SAEC 

**Figure 1:**
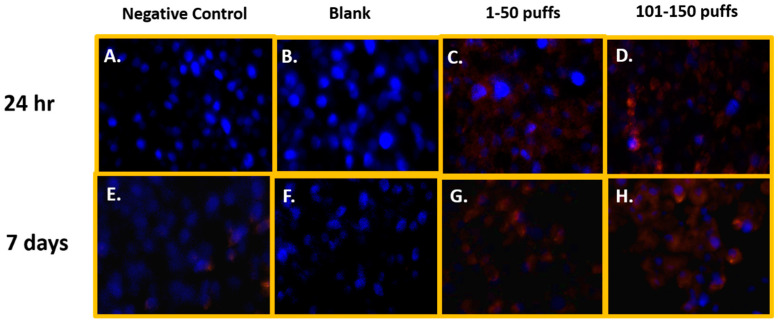
Qualitative ROS generation in SAEC after 24 h and 7 days of ENDS aerosol exposure at two different device-aging stages. At 24 h, Juul Virginia Tobacco exposures elicited significant levels of ROS (**C**,**D**) in comparison to the negative and blank control (**A**,**B**) at both puff fractions 1–50 and 101–150. After 7 days of repeated JUUL exposure, each puff fraction elevated ROS levels (**G**,**H**) in SAEC relative to the negative and blank control (**E**,**F**).

**Figure 2:**
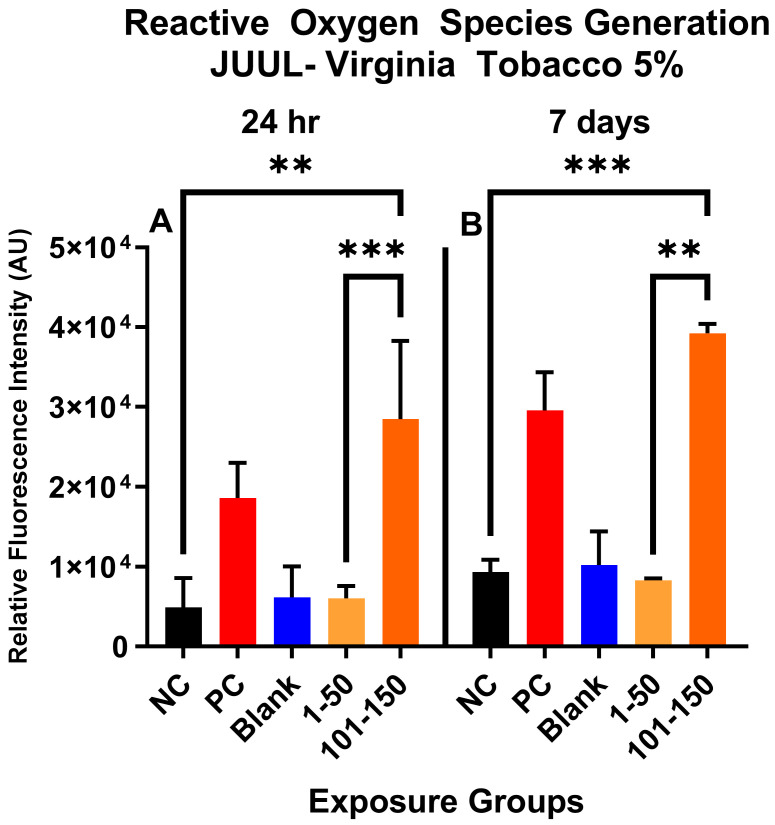
Quantitative data analysis of ROS generation in SAEC after 24 h (**A**) and 7 days (**B**) exposure at two puff fractions (1–50) and (101–150). After 24 h of exposure, there was a significant increase (***, *p* < 0.001) in ROS generation at the later puff fraction (101–150) compared to negative control (**A**). There was significantly higher (**, *p* < 0.01) relative fluorescence intensity detected in the 101–150 puffs than the 1–50 puffs at 24-h (**A**). Similarly, after 7 days of repeated exposure, the later puff fraction (101–150) elicited significantly higher (**, *p* < 0.01) ROS than the 1–50 puff fraction (**B**).

Figure 1 shows the qualitative data for ROS detected in SAEC after 24 h and 7 days of JUUL^®^ Virginia Tobacco 5% exposure at two different concentrations/puff fractions, 39.3 µg/mL (1–50 puffs) and 49.6 µg/mL (101–150 puffs). At 24 h, ENDS-exposed SAEC elicited significant levels of ROS (Figure 1C,D) compared to the negative control (Figure 1A) in both puff fractions 1–50 and 101–150. After 7 days of repeated exposure, each puff fraction elicited elevated ROS levels (Figure 1G,H) in SAEC relative to the negative control (Figure 1E). Figure 2 shows the quantitative data from evaluating multiple areas of several images presented in Figure 1. In Figure 2, a significant increase (*p* < 0.05) in ROS level was noted in the later puff fraction (101–150) after 24 h of exposure, compared to the initial puff fraction (1–50) and negative control (Figure 2A). Similarly, a significant increase (*p* < 0.05) in ROS generation was observed in the later puff fraction (101–150) after the 7 days of exposure in comparisons to the negative control (Figure 2B). Furthermore, a significantly higher increase in relative fluorescent intensity was also detected in the later puff fraction (101–150) compared to the initial puff fraction (1–50) after 7 days of exposure (Figure 2B). No significant changes were observed between the negative control and the blank control in both 24 h and 7 days of exposure (Figure 2). 

### 3.6. ENDS Aerosols Elicit Oxidative Stress

**Figure 3:**
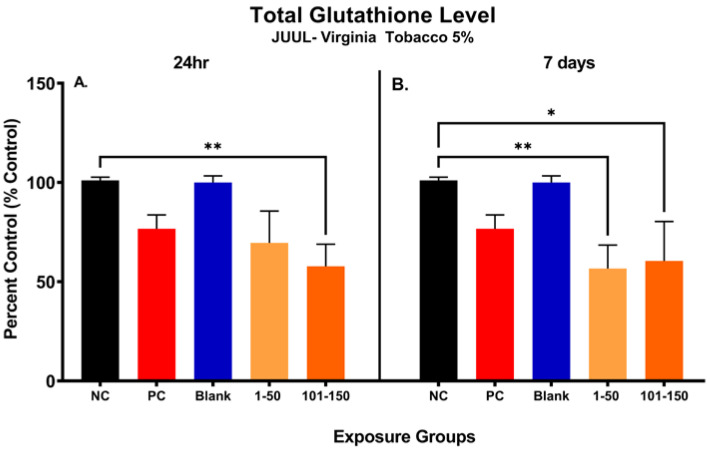
Changes in total glutathione level of pod-type ENDS aerosol exposed SAEC after 24 h and 7 days. The total glutathione level was significantly decreased by 43.25% (**, *p* < 0.01) in 101–150 puffs after the 24 h exposure to JUUL^®^ Virginia tobacco 5% ENDS aerosol compared to the negative control (**A**). Even though there was no significance noted, the total GSH level was decreased by 31.44% also in 1–50 puffs at 24 h (**A**). After the 7 days of exposure, the total GSH level has significantly decreased by 44.39% (**, *p* < 0.01) and 40.50% (*, *p* < 0.05) in 1–50 puffs and 101–150 puffs, respectively, compared to the negative control (**B**). The Blank control, which contained vehicle solution for dilution, showed no difference compared to the negative control (**B**).

Figure 3 shows the changes in total glutathione (GSH) level in SAEC after 24 h and 7 days of exposure at both 39.3 µg/mL (1–50 puffs) and 49.6 µg/mL (101–150 puffs) (Figure 3). Significant reduction (43.25%) in cellular glutathione was observed at later puff fraction (101–150) after 24 h exposure, in comparison to the negative control (Figure 3A). Even though there was no significance noted, the total GSH level was decreased by 31.44% in the initial puff fraction (1–50) after 24 h exposure in comparison to the negative control (Figure 3A). Moreover, the total GSH level was slightly decreased in later puff fraction (101–150) compared to the initial puff fraction (1–50) after 24 h of exposure (Figure 3A). Likewise, after 7 days of repeated exposures, significant reductions in cellular glutathione levels (44.39% and 40.50%) were observed in the initial (1–50) and later (101–150) puff fraction in comparison to the negative control, respectively (Figure 3). However, there was no significant changes noted in the later puff fraction (101–150) in comparison to the initial puff fraction (1–50) in 7 days of exposure (Figure 3B). The blank control, which serves as a method control to account for potential residual extraction solution, showed no difference compared to the negative control (Figure 3B). The cellular glutathione level of the blank sample and negative control remained similar throughout each exposure duration, showing no potential harmful cellular effect of residual extraction solution within samples. 

### 3.7. ENDS Aerosol Exposure Reduce SAEC Metabolic Capacity 

**Figure 4:**
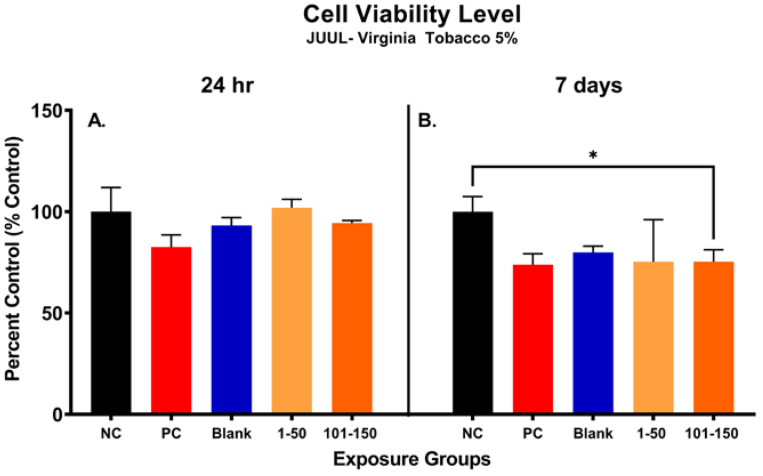
Metabolic capacity of pod-type ENDS aerosol exposed SAEC determined by MTS assay. After 24 h, the metabolic activity of SAEC exposed to JUUL Virginia Tobacco 5% aerosols was not significantly reduced at either of the puff fractions (1–50) and (101–150) (**A**). However, repeated exposure of 7 days elicited a significant reduction (*, *p* < 0.05) in cellular viability of 25% at 101–150 puffs as compared to the negative control (**B**).

Figure 4 shows alterations in metabolic capacity and cellular viability of SAEC after the JUUL^®^ Virginia Tobacco 5% emitted aerosol exposure at 24 h and 7 days. After 24 h, the metabolic activity of SAEC exposed to JUUL aerosols was not significantly reduced at either of the administered doses of 39.3 µg/mL (1–50 puffs) and 49.6 µg/mL (101–150 puffs) (Figure 4A). However, longer exposure duration of 7 days elicited a significant reduction in cellular viability of 25% at the later puff fraction (101–150) as compared to the negative control, indicating a reduction in metabolic capacity (Figure 4B). No significant change in cellular metabolic capacity was noted in the later puff fraction (101–150) in comparison to the initial puff fraction (1–50) after 7 days of exposure (Figure 4B). 

### 3.8. Later Puff Fractions Elicit Higher Levels of Single-Stranded DNA Breaks 

**Figure 5:**
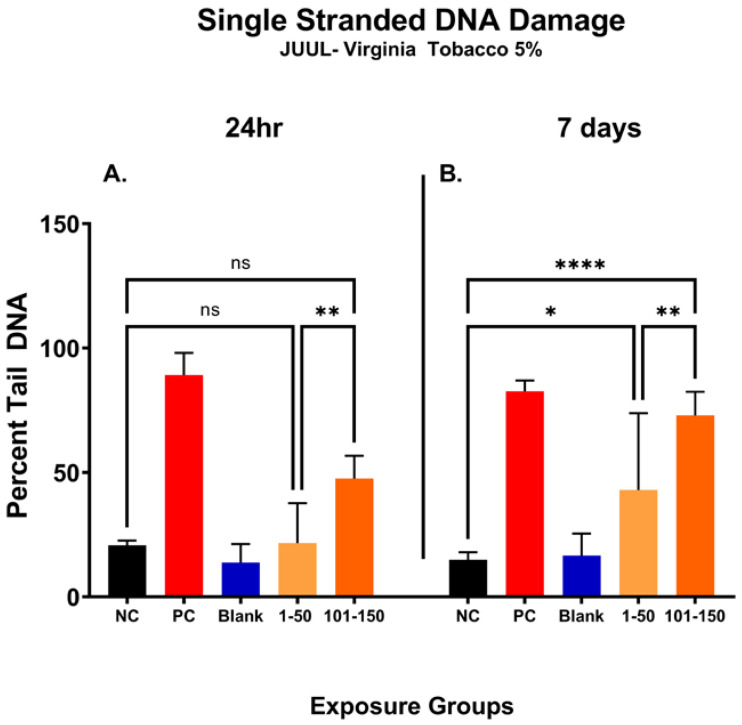
Single-stranded DNA damage of SAEC after 24 h and 7 days of pod-type ENDS aerosol exposure. The percent tail DNA was compared at different puff fractions (1–50) and (101–150) after 24 h and 7 days of exposure to determine the level of DNA damage. After 24 h exposure, there was no significant (ns) change noted at either of the puff fractions, (1–50) or (101–150) (**A**). The longer exposure duration of 7 days showed a significant incline in DNA damage at both puff fractions (1–50) and (101–150) (**B**). At 7 days, the level of DNA fragments in the comet tail has significantly increased by 28% (*, *p* < 0.05) and 58% (****, *p* < 0.0001) in 1–50 puffs and 101–150 puffs compared to the negative control (**B**). Moreover, the later puff fraction (101–150) elicited significantly higher (**, *p* < 0.01) level of DNA damage compared to the initial puff fraction (1–50) at 7 days (**B**).

In Figure 5, the percentage of DNA tail (percent tail DNA) was compared at different puff fractions, 39.3 µg/mL (1–50 puffs) and 49.6 µg/mL (101–150 puffs), after 24 h and 7 days of exposure to determine the level of ENDS-mediated DNA damage. After 24 h, there was no significant change noted in single-stranded DNA damage at either puff fraction (1–50 or 101–150) in comparison with the negative control (Figure 5A). However, the level of DNA damage was significantly increased by 26% at a later puff fraction (101–150) compared to the initial puff fraction (1–50) after 24 h of exposure (Figure 5A). After the longer exposure duration of 7 days, significantly increased levels of DNA damage at both puff fractions (1–50 and 101–150) were observed in comparison to the negative control (Figure 5B). More specifically, the later puff fraction (101–150) at 7 days of exposure elicited 30% more DNA damage than the initial puff fraction (1–50) (58% vs. 28%, respectively) (Figure 5B).

## 4. Discussions

ENDS are advertised as safer alternatives compared to traditional combustible tobacco products, although concerns over their use have continued to grow [38,39]. Recent evidence indicates hazardous chemical compounds are prominent in pod-type ENDS-emitted aerosol, which when characterized within the context of individual vaping frequency or patterns may lead to exposure variances with unknown consequences [7,9]. Numerous toxicological studies have shown inconsistencies that may be attributed to differences in device types, puffing topography profiles, and e-liquid flavorings that were used in each experiment [7,18,40]. Furthermore, there are limited data on the potential hazardous impact of pod-type ENDS related to vaping behavior or patterns, which creates a major knowledge gap regarding the safety of the products that control a significant market stake among ENDS users [6,7,18]. Understanding the mediators of exposure, such as vaping patterns or behavior and device aging, are sorely needed to assess potential health risks associated with pod-type ENDS. This study compared toxicological profiles and elemental properties of JUUL^®^ Virginia Tobacco 5% aerosol in different puff fractions, (1–50) and (101–150), to determine the relative exposure risk of pod-type ENDS devices across the pod lifetime.

In this study, we observed a positive correlation between particle concentration within the aerosol and the device aging process. Furthermore, we also observed a significant size growth and tighter size distribution of the emitted particles as the device ages, which agrees with the measurements from a previous study that observed high particle concentration and submicron-sized particles in the ENDS-emitted aerosol [41,42]. In particular, increasing particle sizes with a narrower particle size distribution can enhance respiratory deposition and sedimentation rate of hazardous particle components within the ENDS aerosol [43,44,45]. For instance, our inhalation dosimetry analysis showed a significant increase in particle mass deposition and deposited surface area as the device ages, which is shown to be highly affected by changes in particle sizes and concentrations. Although there are other factors that may have contributed to particle size growth, such as condensation or coagulation that occurs from minor variations in temperature, relative humidity, and exposure chamber air exchange rates, the changes in particle size distribution observed in this study may also suggest compositional changes of the particles in the emitted aerosols as the device ages [36,46]. Therefore, compositional analysis of the emitted aerosol is essential for further evaluation of the potential health risks of using ENDS devices. 

Among the various chemical components within the ENDS-emitted aerosol, heavy metals are one of the toxicants that can cause critical health effects [11,47,48]. Our findings indicate metal compositional changes that evolve during device usage and aging of components indicating a greater risk of exposure to hazardous levels of toxic metals with continuous use of pod-type ENDS devices. Only two other studies to our knowledge have investigated the difference in aerosolized metal content between new and aged ENDS devices [10,11]. Other studies revealed increasing concentrations of Cr, Zn, Mn, and Ni in ENDS aerosol caused by longer puff durations, which are known to be associated with metallic component degradation during the e-liquid vaporizing process [42,47]. Among these four types of toxic metals, our report only indicates a significant increase in Cr concentration in the later puff fraction (101–150), but not in Zn, Mn, and Ni. Considering that the heating element of the pod-type ENDS device (JUUL^®^) utilized in this study was made of nichrome, our finding aligns with a previous study that showed the significance of the composition of the heating element on the level of metals within the ENDS-emitted aerosol [47]. Moreover, we were able to identify additional types of toxic metals that showed a significant increase as the device ages. Of the 21 metals studied in the current work, six—aluminum, chromium, copper, rubidium, barium, and lead—were found to be significantly (*p* < 0.05) higher within the later puff fraction (101–150) in comparison to puff fraction 1–50. We observed increasing concentrations of manganese and arsenic, but these were not statistically significant. Common metal species such as copper, iron, nickel, and zinc were found to be within ranges of other cartridge-type devices previously reported, most likely leached from the metal heating element as previously described within the plastic pod, and notably, arsenic, barium, and lead concentrations were higher than previously found within the literature [37,49]. This is concerning given that these elements also indicated higher concentrations in the corresponding puff fractions within the context of human exposure. In the framework of human health, the OSHA 8 h exposure level for inhalation represents a standard with which to compare. Copper, zinc, arsenic, and lead concentrations in the current research exceeded acceptable OSHA levels (0.1 mg/m^3^, 15 mg/m^3^, 0 mg/m^3^, and 0.05 mg/m^3^, respectively) for an 8 h exposure period, not only in the later puff fraction but also in the earlier fraction [50]. Chromium and barium concentrations were just below the threshold levels, and aluminum, vanadium, iron, cobalt, and nickel were well below OSHA levels [50]. The concentrations of eight metals studied across the typical life of the device—aluminum, chromium (valency undetermined), manganese, nickel, copper, zinc, arsenic, and lead—even exceeded the time-weighted average threshold limit values (1 mg/m^3^, 0.003 mg/m^3^, 0.1 mg/m^3^, 0.1 mg/m^3^, 0.2 mg/m^3^, 2 mg/m^3^, 0.01 mg/m^3^, and 0.05 mg/m^3^, respectively) set by the American Conference of Governmental Industrial Hygienists (ACGIH) in reference to daily exposure [51]. In puff fractions 1–50, chromium, manganese, nickel, copper, zinc, arsenic, and lead exceeded these ACGIH standards, which is concerning given the variation in puffing behavior for just one session of vaping. In the context of toxicity, the six metals that we found to differ significantly between early and late puff fractions, along with detected manganese, nickel, zinc, arsenic, and strontium, have been found to elicit negative human health consequences at varying levels [11,52]. 

While assessing relevant human exposure levels is critically important in determining the safety of ENDS, understanding various particle reaction dynamics that may occur within biological systems is essential for a holistic appraisal of potential cellular outcomes [53]. One of the reactions that occurs during complex particle-cell interactions is the Fenton reaction, which involves the generation of oxidizing agents such as hydroxyl radicals through the decomposition of hydrogen peroxide in the presence of ferrous cations or other transition metals [54,55]. Among the six metals that our metal analysis detected at an elevated level in the later puff fraction (101–150), three transition metals (Cu, Cr, and Al) are considered Fenton reagents. Fenton reagents can induce Fenton-like reactions and increase reactive oxygen species (ROS) production [55,56]. This correlates with our toxicological observations, which showed elevated ROS caused by later puff fraction exposure, suggesting metal-induced oxidative stress. While we noted substantial levels of ROS in the later puff fractions, similar levels of ROS were not observed in earlier puff fractions although decrements in SAEC glutathione levels that were due to each puff fraction exposure were similar across both timepoints. This discrepancy could potentially be due to the presence of other free radicals such as nitric oxide and peroxynitrite that are potent oxidative stress inducers within the airways [57]. Furthermore, other hazardous aerosol components such as semi-volatile organic compounds (sVOCs), which were not assessed in our aerosol extracts, may have contributed to the reduction in total glutathione levels observed in ENDS exposed SAEC [58]. With regard to copper, recent studies have observed elevated ROS production and cellular injury because of increasing copper particle concentrations in the ENDS aerosol [59,60]. Copper particles are concerning because of their high chemical reactivity that can induce DNA fragmentation in respiratory cells [60,61]. We observed a significant association between increases in Cu and other metal concentrations and DNA damage in the later puff fraction (101–150) [58]. Likewise, chromium, which can exist in several oxidation states, can elicit DNA damage, cell transformation, and impaired lung function upon exposure [62]. Aluminum, which was greatly elevated because of device ageing in our assessment, can accumulate within lung fluids upon inhalation and lead to respiratory disease [63]. 

Importantly, other factors such as reactive nitrogen species and sVOCs, which were not assessed in this study, may have contributed to the observed levels of DNA damage elicited by both puff fractions. However, our findings are consistent with recent epidemiological evidence revealing ENDS users have significantly higher levels of metals and oxidative DNA damage than non-vapers [64]. Importantly, others have theorized that the ENDS-related metal aerosols can contribute to poor indoor air quality and cause metal bioaccumulation within respiratory tissues leading to disease [65]. The specific pathways or mechanisms of action leading to respiratory disease or altered function have yet to be verified but certainly warrant further exploration. Our findings, along with others, underscore the potential hazardous biological effects of metal toxicants emitted from aged pod-type ENDS devices [1,32]. It should be noted that the JUUL^®^ pod manufacturer claims their products contain sufficient e-liquid for 200 puffs; thus, a user may vape over 150 puffs using a single pod. In the case of the “dry-puff” scenario, increased emissions and potentially more hazardous exposure could occur [66]. This highlights the need for further toxicological analysis of other chemical components in the ENDS aerosol, such as volatile organic compounds (VOCs), to determine additional health risks that ENDS usage may pose [67,68]. 

## 5. Limitation

In this study, we investigated the cellular responses of pod-type ENDS aerosol exposures to explore the relationship between device ageing, metal emission composition, and respiratory impacts using a small airway epithelial in vitro model. Although the selected pod-type ENDS device, JUUL^®^, was one of the most popular brands at the time when the study was conducted, the ENDS market is changing rapidly with emerging popular pod-type devices. More studies on other brands of pod-type ENDS devices are needed for generalizable conclusions on the health impacts of pod-type ENDS devices. In addition, this study was designed for laboratory characterization and analysis, based on the research capacity and instrumentation availability at the time when the study was conducted. Specifically, aerosol components such as sVOCs that may have contributed to overall toxicological observations were not assessed within the aerosol extracts. While we aimed to simulate real-life vaping profiles using CORESTA Method 81, which uses a standardized user’s puff topography, we acknowledge this may not encompass all vaping scenarios. Additionally, we defined the maximum puff number for a pod to be 150 based on our optimization studies and recent literature to avoid the “dry-puff” or coil heating without adequate e-liquid. However, we recognize this approach does not account for differences in vaping frequency caused by user preferences. Lastly, we note that comprehensive assessment of ENDS metal-exposure health impacts is determined through empirical and epidemiological approaches where urine or blood samples are employed; however, this was not the scope of this current study. In our future work, we will be collecting and analyzing biosamples from human subjects to further understand the health impacts of ENDS exposure. 

## 6. Conclusions

The prolonged use of pod-type ENDS devices, or device aging, may contribute to greater exposure risks to hazardous aerosols and metals resulting in enhanced oxidative stress, cellular viability reduction, and DNA damage in exposed SAEC. As the pod-type ENDS device ages, increasing concentrations of hazardous metals were produced, including chromium, copper, and lead, which may contribute to the additional toxicity of pod-type ENDS-emitted aerosol. The observed in vitro cellular toxicological effects of ENDS aerosols and metals including the generation of ROS, reduction in total glutathione level, decreased cellular viability, and increase in DNA damage on SAEC indicate potential adverse respiratory effects. This study suggests that continuous use across the lifetime of pod-type ENDS and over extended periods may increase the risk of hazardous metal exposures. These findings underline the need for further investigation into device aging and vaping behavior of pod-type ENDS users to determine associated pathophysiological consequences and device safety. Therefore, the clinical impact of the differential metal and chemical exposures derived from ENDS usage on oral health will be discussed in our subsequent study.

## Figures and Tables

**Table 1:** Pod-type ENDS-emitted aerosol concentration and mean diameter at different puff fractions.

Pod-Type Tobacco e-Liquid with 5% Nicotine		
Puff Fraction	Particle Conc. (mg/m^3^)	GMD (µm)
1–50	0.00212 ± 0.0018	0.715 ± 0.322
101–150	0.135 ± 0.0586	0.902 ± 0.0272

**Table 2:** Metrics of ENDS Aerosol Deposition within Human Lungs.

Puff Fraction	Average Total Deposited Mass (µg)	Average Deposited Surface Area (cm^2^)	Average Deposited Dose (µg/cm^2^)	In Vitro Concentration (µg/mL)
Pod-type Tobacco e-liquid with 5% nicotine				
1–50	2.66 × 10^−7^	2.23 × 10^−8^	1.19 × 10^1^	39.3
101–150	1.52 × 10^−5^	1.01 × 10^−6^	1.50 × 10^1^	49.6

**Table 3:** Average amounts (*n* = 2, σ < 1) of metals of concern to human health in tobacco-flavored e-liquid and resulting ENDS aerosol puff fractions.

		Puff Fraction	
	e-Liquid (µg/L)	1–50 (µg/L)	101–150 (µg/L)
**Al**	ND	0.109	2.147
**V**	0.016	<LOD	0.016
**^52^Cr**	0.259	0.157	0.306
**^54^Fe**	0.982	ND	ND
**Mn**	0.010	0.167	0.180
**^56^Fe**	1.179	0.283	<LOD
**^57^Fe**	1.231	0.402	<LOD
**Co**	0.014	0.006	0.004
**Ni**	0.693	0.124	0.111
**^63^Cu**	0.353	8.109	12.241
**^65^Cu**	0.353	8.112	12.386
**Zn**	0.702	45.046	44.465
**As**	0.063	0.050	0.052
**Rb**	ND	0.012	1.210
**Sr**	0.036	0.126	0.111
**Ag**	ND	ND	ND
**Cd**	<LOD	<LOD	<LOD
**Ba**	0.063	0.006	0.262
**Tl**	ND	<LOD	<LOD
**Pb**	0.032	0.546	1.161
**U**	ND	ND	ND

Underlined values are significantly different (*p* < 0.05) between puff fractions. Concentrations were given in the same units to simplify comparison. Less than the limit of detection (<LOD) refers to an instrument response above zero concentration but below the calculated instrument detection limit. Not detected (ND) refers to a negative or zero concentration.

## Data Availability

The data presented in this study are available upon request from the corresponding author.

## Supplementary Materials

The following supporting information can be downloaded at https://www.mdpi.com/article/10.3390/toxics11020155/s1: Table S1: Summary of parameters used in the Multiple Path Particle Deposition model for deposition modeling of ENDS aerosols; Table S2: ICP-MS Tuning and Operating Parameters; Table S3: Internal standard compatibility.
