# Supplementary material for: Toxicological Assessment of Particulate and Metal Hazards Associated with Vaping Frequency and Device Age
Source: Toxics. 2023 Feb 7;11(2):155. doi: 10.3390/toxics11020155 (PMC9967192; doi:10.3390/toxics11020155)
Supplement: Supplementary file 1 [file toxics-11-00155-s001.zip › toxics-2130647-supplementary.pdf]

Supplementary Material

Table S1. Summary of parameters used in the Multiple Path Particle Deposition model for deposition modeling of ENDS aerosols.

Human Stochastic Model	***Breathing Parameters***	***Exposure Parameters***
Functional Residual Capacity: **3300 mL**	*Tidal Volume:* **625 mL**	*Number of Hours/Day:* **6**
Head Volume: **50 mL**	*Breathing Frequency:* **12 breaths/ min**	*Number of Days/Week* **7**
Breathing Scenario: **Oral**	*Inspiratory Fraction:* **0.5** *Pause Fraction:* **0.0**	*Number of Weeks:* ***52***

ICP-MS Analysis Parameters

The Nexion 5000 ICP-MS was equipped with an autosampler (SC2-DX, 2DXCi, Elemental Scientific) and FAST sample introduction system (Elemental Scientific). A manufacturer-recommended tune was performed on the day of analysis that monitored elemental intensities, oxide ratio, background concentration, torch alignment, voltages, and flow rates using a tuning solution consisting of Be, Ce, Fe, In, Li, Mg, Pb, and U (200 ng/L, Perkin Elmer). The optimal instrumental conditions after setup that were used for analysis are outlined in Supplemental Table S2.

All reagents utilized in the analysis were made using a Barnstead E-Pure Ultrapure water purification system that generated Type I ultrapure water. A 2% nitric acid solution (prepared with 60-70% Aristar Plus Trace Metal Nitric Acid, VWR, Lot#1121070) was used for all tubing rinses, carrier probe solution, and standard and sample dilutions. For preparation of the e-liquid samples and aerosol samples following that outlined in the Methods section above, the sample tubes were shaken repeatedly to mix and allowed to settle before analysis.

Two types of quality control measures were used to ensure correct quantitation of elemental species: external calibration and internal calibration. All calculations were performed by the ICP-MS software suite (Syngistix, Perkin Elmer). The external calibration and curve calculations were performed prior to sample analysis and consisted of analyzing a five-point curve (0.1 µg/L, 1 µg/L, 5 µg/L, 10 µg/L, and 20 µg/L) of the following elements: Ag, Al, As, Ba, Be, Bi, Ca, Cd, Co, Cr, Cs, Cu, Fe, K, Mg, Mn, Na, Ni, Pb, Rb, Se, Sr, Tl, U, V, and Zn (IV-STOCK-21, 10 µg/mL stock concentration, Inorganic Ventures). Internal calibration was utilized to ensure matrix effects were minimized and to provide another measure of confidence in the results. An approximately 200 µg/L internal standard (IS) solution was prepared using a mixed stock solution of 10 µg/mL of the following elements: Bi, In, ^{6}Li, Sc, Tb, and Y (IV-ICPMS-71D, Inorganic Ventures). Each metal of interest in the study was matched to the appropriate internal standard by closest atomic mass unit with few exceptions, outlined in Supplemental Table S3.

All coefficients of determination (R^2) for the elements of interest in the study were greater than or equal to 0.98. Additionally, a calibration check standard sample (5 µg/L) was analyzed within the run about every ten to fifteen samples (depending on order of sequence) to monitor drift. The RSD values among three replicates of each element reported were less than or equal to 21% except for Fe, Cd, and Tl. If quantitation values were below the lowest calibrator, they were not reported in the results, and if the sample gave a value higher than the highest calibrator, the sample was diluted and rerun with an inferred dilution calculation. Limits of detection (LOD) and quantitation (LOQ) were calculated within 30 days of the study analysis using seven replicates each of blank and 1 µg/L calibrator solution for all metal species of interest using the customary 3σ and 10σ for LOD and LOQ, respectively.

Table S2. ICP-MS Tuning and Operating Parameters

Parameter	Setting
Tuning parameters	^{9}Be > 2500 cps<br>^{115}In > 90000 cps<br>^{238}U > 55000<br>Bkgd/220.5 ≤ 3<br>CeO/Ce < 0.03
RF power	1600 W
Ar plasma flow	16 mL/min
Ar auxiliary flow	1.2 mL/min
Nebulizer flow	1.02 mL/min
Cell gas flow (He)	3.5 mL/min
Peristaltic pump speed	-18 rpm
Sampling and skimmer cones	Nickel
Sampling depth	0.579 mm
Detector/Detection mode	Dual / MS/MS
Dwell time	50 ms
Sweeps	20
Replicates	3
Flush; read delay; wash	15 s; 20 s; 17 s

The parameters of optimal instrumental condition of Perkin Elmer NexION 5000 ICP-MS with an autosampler (SC2-DX, 2DXCi, Elemental Scientific) and FAST sample introduction system (Elemental Scientific) is outlined above (Supplemental Table S2). Prior to analysis, a manufacturer-specified performance check was performed and the elemental intensities, oxide ratio, background concentration, torch alignment, voltages, and flow rates were monitored by using a tuning solution consisting of Be, Ce, Fe, In, Li, Mg, Pb, and U (200 ng/L, Perkin Elmer).

Table S3. Internal standard compatibility

**Internal Standard Element**	**Metal of Interest**
^{6}Li	Be, Na, Al, K
Sc	Mg, Ca, V, Cr, Fe, Mn, Co, Ni, Cu
In	Zn, As, Se, Ag, Cd
Y	Rb, Sr
Tb	Cs, Ba
Bi	Tl, Pb, U

The table above outlines the list of metals of interest and their respective internal standard elements that were selected for the internal calibration. The internal calibration provides an additional measure of confidence in the analysis result of this study and ensures matrix effects are minimized. An approximately 200 µg/L internal standard (IS) solution was prepared using a mixed stock solution of 10 µg/mL of the following elements: Bi, In, 6Li, Sc, Tb, and Y (IV-ICPMS-71D, Inorganic Ventures).
